# Unilateral Subfrontal Approach for Giant Tuberculum Sellae Meningioma: Single Center Experience and Review of the Literature

**DOI:** 10.3389/fonc.2021.708235

**Published:** 2021-08-09

**Authors:** Feng Xiao, Jie Shen, Luyuan Zhang, Jiqi Yang, Yuxiang Weng, Zebin Fang, Chao Zhang, Hongxing Ye, Renya Zhan, Xiujue Zheng

**Affiliations:** Department of Neurosurgery, The First Affiliated Hospital, Zhejiang University School of Medicine, Hangzhou, China

**Keywords:** giant tuberculum sellae meningiomas, frontal sinus repair, unilateral subfrontal approach, optic canal decompression, microsurgical transcranial approach

## Abstract

**Background:**

Microsurgical Transcranial approach (mTCA) is the primary choice for the resection of giant Tuberculum Sellae Meningiomas (TSM). The objective of this study is to explore surgical details of unilateral subfrontal approach.

**Methods:**

Ten patients with giant TSM treated by unilateral subfrontal approach were included from January 2018 to June 2021. Demographic characteristics, surgical data, post-procedure complications and outcomes of patients have been descriptive analyzed, combined with systematic literature review to explore the surgical details and the prognosis of unilateral subfrontal approach.

**Results:**

Ten patients include six male and four females, age range from 35 to 77 years, duration of visual impairment from 1 to 12 months, were all performed unilateral subfrontal approach. Nine patients achieved radical resection (Simpson grades I-II) through post-operative imaging confirmation, and Simpson IV resection was performed in the remaining one due to cavernous sinus invasion. The postoperative visual acuity was improved or maintained in 8 patients. Visual acuity decreased in 2 cases, including 1 case of optic nerve atrophy and the other case of optic canal not opening. Five cases with frontal sinus opened were repaired during the operation and there was no postoperative cerebrospinal fluid leakage or intracranial infection. One patient suffered from postoperative anosmia, one patient developed left limb weakness, but their symptoms have improved in the follow-up.

**Conclusion:**

Summarize the experience of our center and previous literature, unilateral forehead bottom craniotomy is a feasible surgical approach for giant tuberculum sellae meningioma. Intraoperative application of EC glue and pedicled fascia flap to repair the frontal sinus can prevent complications associated with frontal sinus opening. Optic canal unroofing has huge advantage in visual improvement.

## Introduction

Meningiomas are the most common tumors arising from the meninges by far, accounting for 13%-26% in primary intracranial tumors, more common among female (1, 2). The clinical manifestation and course of disease may be quite different on individuals, a portion are characterized by asymptomatic lesions with slow growth which found by incidental computerized tomography, some present a headache, but sometimes, the progressive enlargement of tumors may lead to seizures, neurological deficits occurred after adjacent nerve tissues compressed, disorder of hormone secretion caused by impairment of pituitary (1, 3). Tuberculum sellae meningiomas (TSM) approximately account for 5%-10% in all meningiomas, occur in the sphenoidal platform, anterior bed, saddle nodule, and saddle septum (3–7). Microsurgical resection is still the first choice for tuberculum sellae meningioma. The anatomy location of giant TSMs (maximum diameter ≥ 3cm) are deeper and more complex, some of giant TSMs often wraps around optic nerve, internal carotid artery and branches, oculomotor nerve, pituitary gland and pituitary stalk (8). As a result, the difficulty of total resection of the tumor is greatly increased, and postoperative complications are difficult to completely avoid. There are still great challenges in clinical practice on how to achieve the maximum excision of tumors, protect blood vessels and nerves, and reduce postoperative complications. Microsurgical craniotomy has always been the main surgical method for the treatment of tuberosity sellae meningioma due to its good total tumor resection rate, however, it also has disadvantages such as large surgical trauma and easy traction of the optic nerve. We present a case series of 10 patients with giant tuberculum sellae meningiomas and summarize literature to explore the surgical details of unilateral subfrontal approach.

## Materials and Methods

### Study Population

Patients diagnosed with TSM and treated by microsurgical transcranial resection between January 2018 to June 2021 in Department of Neurosurgery, The First Affiliated Hospital, College of Medicine, Zhejiang University were included in this case series report. Of 39 patients with TSM in our center, 10 patients met the following inclusion criteria: MRI showed that a space-occupying lesion with a diameter of more than 3 cm was observed in the saddle nodule; Postoperative pathology of tumor revealed meningioma. Written informed consent was obtained from all individual participants before procedures and permission for the publication. Giant TSM were all resected by the same surgeon using unilateral subfrontal approach. Patients whose giant TSM resected by other surgical approaches were excluded, and also those absence of important medical information.

### Clinical Therapeutic Protocol

**Figure 1:**
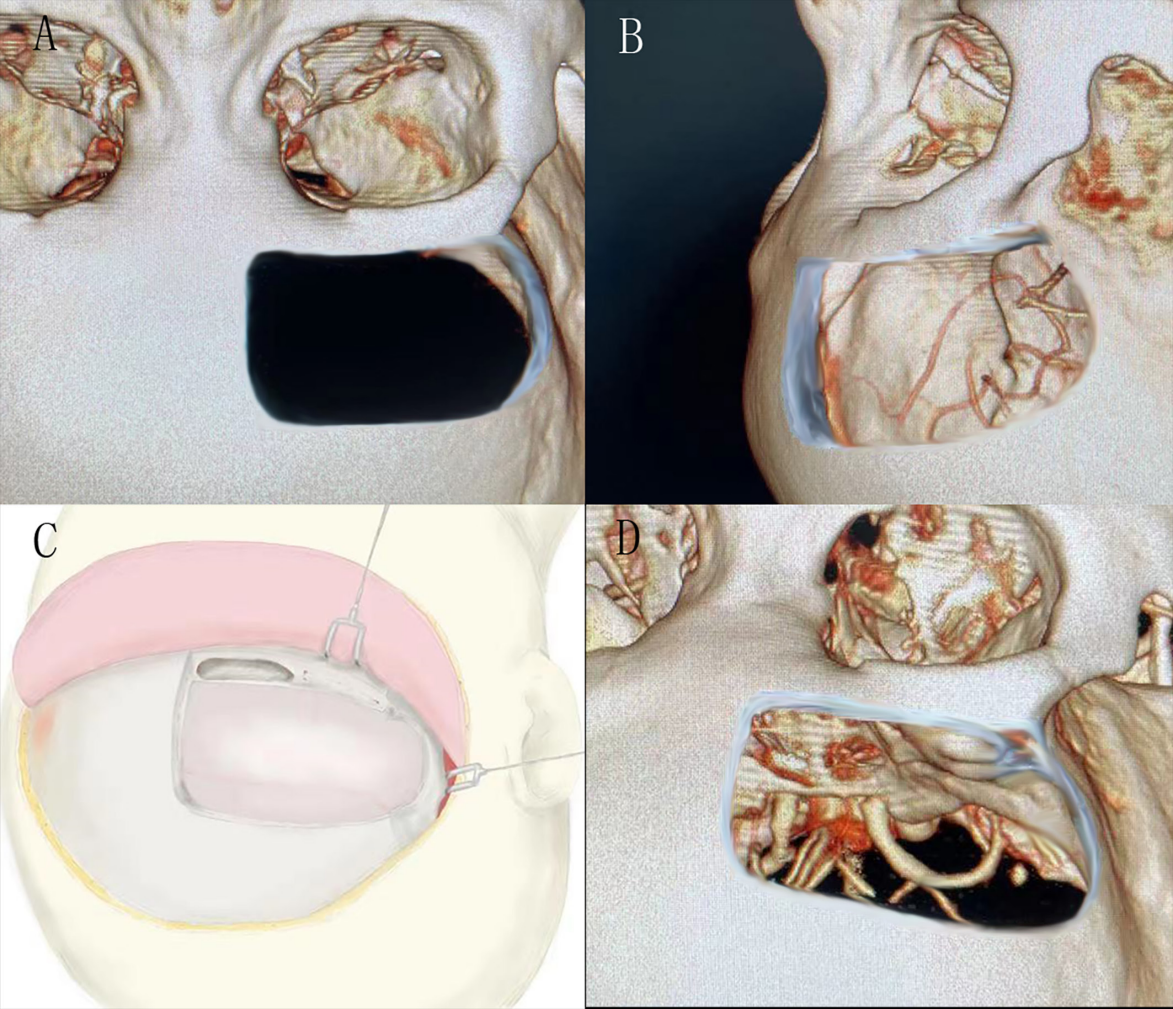
**(A)** and **(B)** showed the bone window of the unilateral subfrontal approach, the larger extension to the temporal side, the more fields of surgical view exposed. **(C)** showed the schematic diagram of unilateral frontal floor craniotomy. **(D)** showed the visual field of sellar area by unilateral subfrontal approach.

**Figure 2:**
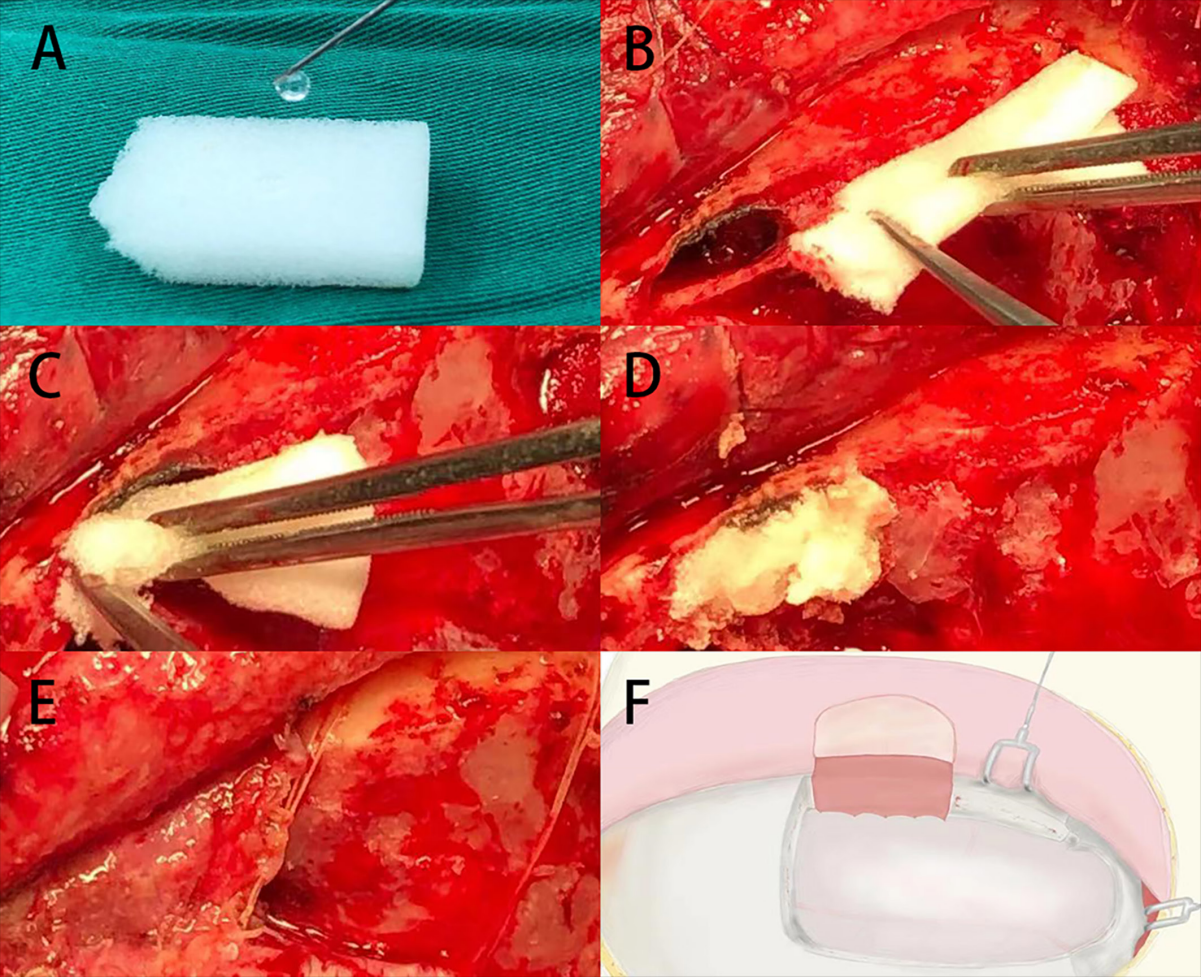
**(A)** The gelatin sponge was dripped with EC glue. **(B–D)** The open frontal sinus cavity was filled with gelatin sponge containing EC glue. **(E, F)** The open frontal sinus was covered by subcutaneous pedicle and sutured with the frontal meninges.

All patients’ meningiomas were resected by the same experienced surgeons using unilateral subfrontal approach (**Figure 1**). The repair process of frontal sinus is as follows (**Figure 2**): Ear-cerebrum (EC) glue was dripped into the gelatin sponge and filled the open frontal sinus cavity with gelatin sponge containing EC glue. Finally, the open frontal sinus was covered by subcutaneous fascia of the forehead and then sutured with the meninges. Optic canal unroofing was taken in patients with tuberculum sellae meningiomas which invaded optic canal.

### Clinical Variables and Definition

The demographic features of the patients, symptoms upon admission, duration of visual impairment, visual acuity and visual field, surgical approaches, intraoperative video, size of meningiomas are reviewed and described. Some potential postoperative complications such as anosmia, cerebrospinal fluid (CSF) leakage, cerebral infection, epilepsy, artery injury, endocrine hormones disorder and so on were carefully examined in every patient. Anosmia was tested using the UPSIT (9). Odor identification refers to a person’s ability to produce and attach a verbal label to an odor, or to identify an odor that matches a verbal or nonverbal (picture) label provided by another person (10).

### Outcome Measures

Postoperative magnetic resonance imaging was the preferred investigation of choice to assess the gross resection rate of tumors, residual and recurrence of tumor. The extent of meningioma resection was assessed by Simpson classification criteria (11). The visual acuity prognosis was assessed by a neurosurgeon using visual acuity chart on discharge and three months after discharge *via* telephone call or outpatient appointment.

## Result

**Figure 3:**
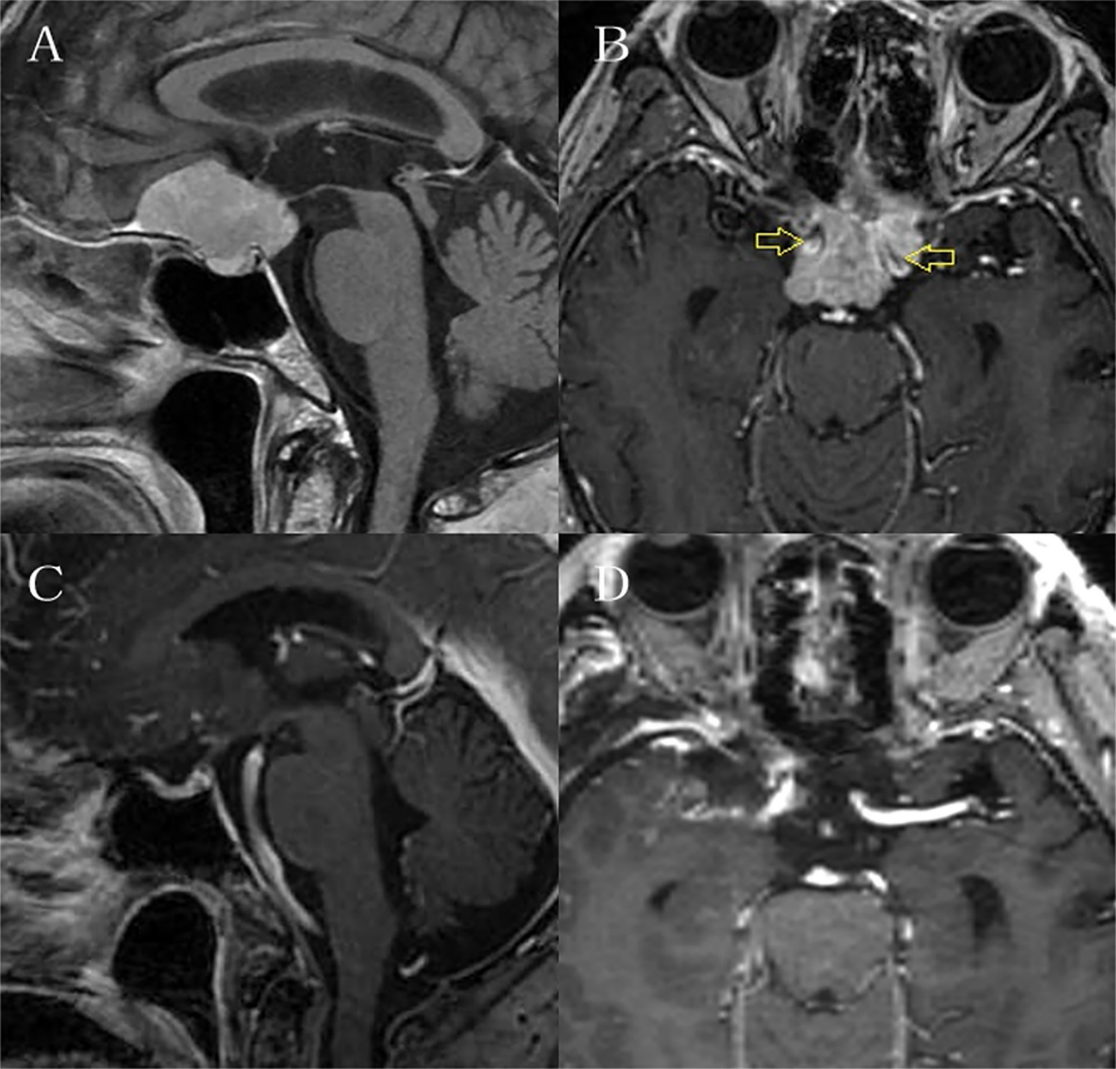
It is the preoperative and postoperative MRI enhancement figure of one case. **(A)** and **(B)** showed the tumor was huge and surrounded bilateral internal carotid arteries. **(C)** and **(D)** showed total resection of the tumor.

**Figure 4:**
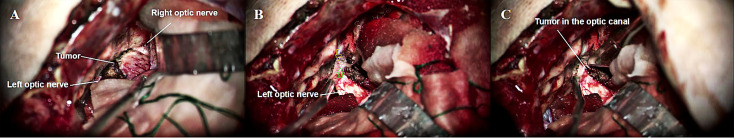
**(A)** Exposure of bilateral optic nerves and tumors *via* left subfrontal approach. **(B)** Open the left optic canal (Yellow arrow) and the left falciform ligament (Green arrow). **(C)** The left falciform ligament was incised to expose the optic canal tumor.

Ten cases of TSM were included, including 6 males and 4 females (**Table 1**), with an average age of 54 ± 3.7 years old. All of them were giant TSM with maximum diameter (calculated according to the maximum diameter of sagittal MRI tumor) ≥ 30mm, with an average size of 34.3 ± 1.03 mm. **Table 1** summarizes the patients’ characteristics. Simpson I-II resection was performed in 9 cases (**Table 1**, **Figure 3**). Neurosurgeon resected the main tumors and the meningeal invasion site during the operation and cauterize the tumor attachment site by electrocoagulation at the same time. The remaining 1 case underwent Simpson IV resection because the tumor invaded the cavernous sinus and grew into the cavernous sinus. There was still residual tumor in cavernous sinus after operation. Eight patients’ optic canal were invaded by the tumor, all of them performed unilateral optic canal unroofing and enlargement, incised falciform ligament and resected peripheral tumors of optic nerve (**Figure 4**). And all of these eight patients had visual acuity improved after operation. Two cases suffered varying degrees of vision loss: One case had only preoperative light perception, the tumor was huge and the optic nerve was obviously compressed and pushed and thinner than normal, these suggested that optic nerve atrophy; Another case may have optic canal invasion, but the optic canal was not unroofed during the operation. The frontal sinus was opened in 5 cases during the operation, all of which were filled and repaired with EC glue, and covered with frontal subcutaneous pedicled fascia flap (**Figure 2**). There was no cerebrospinal fluid leakage and intracranial infection in these 5 patients. In one case, surgeon was obliged to stretched the frontal lobe due to the huge tumor. As a result, there was extensive edema of the frontal lobe after the operation, and the muscle strength of the left limb decreased. After 6 months of rehabilitation, patient’s muscle strength of the limb improved. In another case, transient loss of smell occurred after the operation, and the olfactory function was improved after 6 months follow-up.

**Table 1:** Clinical characteristics of patients and tumors.

Case	Gender	Age (year)	DVI (month)	Size/mm	Simpson Grade	VOA	Complications
1	M	35	Normal	33	I	stable	
2	M	46	Normal	32	I	stable	
3	F	67	1	32	I	improved	
4	M	77	12	39	II	stable	left limb weakness
5	M	54	3	30	II	stable	anosmia
6	M	55	6	31	I	stable	
7	F	48	10	37	II	right deteriorated, left stable	
8	F	48	2	39	IV	improved	
9	F	57	6	36	II	right stable, left deteriorated	
10	M	53	1	34	II	stable	

DVI, Duration of visual impairment; VAO, Visual sympton after operation.

## Discussion

TSM is a general term for meningioma originating from the skull base and the saddle area transition zone. Since Cushing completed the first resection of tuberculum sellae meningioma in 1916, surgery has been the preferred treatment for tuberculum sellae meningioma (1, 5, 6, 12). The surgical approach for tuberculum sellae meningioma mainly depends on the size, growth direction and neurovascular invasion of tumor. At present, the most commonly used surgical approaches include subfrontal approach (13), anterior interhemispheric approach (14, 15), pterional approach (16, 17), supraorbital keyhole approach (7, 18), and endoscopic endonasal approach (EEA) (12, 19). There are many disputes about the choice of surgical approach at present, but microsurgery is the key to totally remove tumors.

Unilateral or bilateral subfrontal approach was usually selected in earlier microsurgery to resect TSM (13). The subfrontal approach can provide a wide and direct field of vision, clearly expose bilateral optic nerve, internal carotid artery, anterior cerebral artery and anterior communicating artery to protect visual nerve and adjacent vessels, facilitate the reconstruction of anterior cranial fossa base. In a long-term follow-up study of subfrontal approach for TSM patients (13), radical resection (Simpson Grades I and II) was achieved in 79% of patients and 91% of patients with preoperative visual impairment showed significantly improved postoperative visual impairment score (VIS). In previous literature, bilateral subfrontal approach can achieve visual improvement more effective than other surgical approaches (6, 13, 20). Unilateral subfrontal approach can expose bilateral optic nerves at the same time and perform decompression of bilateral optic canal, there is no obvious disadvantage compared with bilateral frontal floor approach. Although the visual field of unilateral subfrontal approach is smaller than that of bilateral frontal floor approach, the scope of craniotomy is smaller and the surgical trauma is smaller. And there is no need to ligate the sagittal sinus, the contralateral olfactory nerve can also be reserved, so the possibility of postoperative olfactory dysfunction is reduced. In our case series, one patient had postoperative olfactory loss, but then improved after discharge.

Pterional approach is a classic approach for skull base surgery. Studies have shown that pterional approach is more suitable for sellae meningiomas with tumors growing on one side and posterior sellae region (5, 16, 17). The pterional approach has three main advantages: First, it can minimize the damage to the olfactory nerve through fully exposing of the ipsilateral olfactory nerve without cutting off the olfactory tract. Second, the frontal bone window edge is generally far from the frontal sinus, and the risk of cerebrospinal fluid leakage or frontal sinus infection is low. Third, this approach has the shortest distance to the sellae region. However, a large number of huge sellae tubercle meningiomas often adhere to the optic nerve and invade the optic foramen, which makes pterional approach for resection of sellae tubercle meningiomas prone to residual (21). And ipsilateral optic nerve and inferior surface of the optic chiasma cannot be fully exposed, previous literature shows that about 10% to 20% of patients with pterional approach may have visual impairment after surgery (22–26). However, by using a unilateral frontal approach, attaining a fuller anatomy exposure on optic nerve areas, the inferior surface of the optic nerve can be clearly visualized under the microscope and its perforators can be carefully preserved. Thus, the improvement of visual function is better in unilateral frontal approach. In addition, unilateral frontal approach has more widely vision of sphenoid plateau and sellae tubercle compared with pterional approach, and its field of obtained vision during the operation is also wider. At the same time, unilateral frontal approach is more beneficial for the protection of blood vessels in the sellae area because of the clearly exposure of the lateral field of internal carotid artery.

Anterior interhemispheric approach was first used in anterior communicating artery aneurysm clipping (27). With the progress of microsurgery, it has become an alternative surgical approach for tuberculum sellae meningiomas (7). It provides a broad and direct surgical approach to the entire optic nerve apparatus and its surrounding structures, including the tubercle sellae, and without excessive frontal lobe contraction. Current literature has reported that the gross total resection (GTR) of this approach for tuberculum sellae meningiomas is over 90% (14, 15). In addition, the interhemispheric fissure approach has more visual enhancement benefits, at least, fewer visual deterioration after surgery (28). And without opening the frontal sinus during surgery, cerebrospinal fluid leakage is also rare. The shortcomings of this approach is that surgeon require more elaborate micromanipulation and longer operative time to dissect the anterior hemispheric fissure and more chance to occur anosmia after interhemispheric approach (28). Moreover, the longitudinal fissure approach is relatively difficult to expose the lateral side of the tumor compared with the unilateral subfrontal approach.

The endoscopic endonasal approach has become a new alternative technology different from transcranial approach (3, 29, 30). EEA offers several advantages over TCA, such as maximize the removal of all bone and dura involved by tumor; less manipulation of the optic nerves, optic chiasm, and brain; improved visualization of the medial optic canal and without skin incision, more aesthetically pleasing (31–34). However, in the early period, the outcome of tuberculum sellae meningiomas resected by EEA is not satisfactory. Prior to 2012, a meta-analysis comprised 60 series and 1426 patients revealed the GTR of TSM was higher in patients undergoing microsurgical transcranial surgery compared those undergoing EEA (84.1% *vs* 74.7%; p = 0.041) (35). In another meta-analysis included 64 case series which published before 2017, found no significant statistical significance in GTR between mTCA and EEA for patients with TSM (EEA: 83.0% *vs* mTCA: 85.8%, p = 0.34), however, visual improvement was higher in EEA than mTCA for patients with TSM (77.7% *vs* 60.7%, p < 0.01) (12). The mainly advantages of EEA are the protection of optic nerve and the improvement of visual acuity after surgery, but its main problems of EEA are the high incidence of cerebrospinal fluid leakage and meningitis. Most of the current studies consistently confirm that EEA provides better results at the expense of higher CSF leakage rates in visual restoration and preservation (12, 13, 34–36). It is worth mentioning that the risk of cerebrospinal fluid leakage after EEA has been gradually reduced with the improvement and standardization of reconstruction techniques of nasal septal flaps (37, 38). The prominent drawback of EEA is difficult to resect giant tumor (the largest diameter ≥ 30mm), tumor with tough texture or the lateral growth tumor that invades the optic canal and surrounds the internal carotid artery. Therefore, craniotomy is still the first choice for giant complex tuberculum sellae meningiomas.

Unilateral subfrontal approach requires fully exposing the base of anterior cranial fossa to reduce the traction of the frontal lobe, so the frontal sinus will be opened in most cases. Some researches pointed out the potential risk of cerebrospinal fluid leakage, meningitis, olfactory nerve injury caused by intraoperative frontal sinus opening, and the chance of venous infarction caused by occlusion of the superior sagittal sinus (6). Nevertheless, these complications are not specific to subfrontal approach and, indeed, also exist in the series reports of other surgical approach. And in fact, as long as a better exposure of sellae structure, frontal sinus could be repaired with free fat tissues, the effect of preventing CSF leakage and frontal sinus infections were satisfactory (39, 40). Opening the frontal sinus combined with preoperative navigation to determine the path of the milling cutter to ensure the integrity of the frontal sinus mucosa is a reasonable option. However, it is difficult to completely preserve the frontal sinus mucosa in cases where the frontal sinus is well developed and the top is taller and wider. The procedure of frontal sinus repair in our center is as follows: frontal sinus mucosa was separated and pushed downward during the operation; EC glue was dripped into the dry gelatin sponge and tightly packed in the open frontal sinus orifice; finally, the subcutaneous tissue of the frontal scalp was taken to form a pedicled tissue flap to cover the open frontal sinus orifice, and sutured with the meninges on the inner side of the skull. No cerebrospinal fluid rhinorrhea or frontal sinus associated infection occurred in all cases.

The optimal surgical goal for tuberculum sellae meningioma is to resect as much tumors as possible while preserving good neurological function. In this case series, 9 cases of tumor achieved Simpson I-II resection, and no obvious residual was found on MRI after 3 months of operation, which shows that unilateral subfrontal approach will not reduce the total resection rate of tumor. The effectiveness of radiotherapy as an adjuvant therapy for remnant meningiomas has already been confirmed (41–43). Therefore, when we could not attain gross total resection for tumor, it is more meaningful to preserve the neural function and ensure the self-care ability of patients. According to statistics, the proportion of vision deterioration after surgery for tuberculum sellae meningioma can reach 10 to 40%. Several influence variables of postoperative visual outcome include tumor size (44), the site and extension of the tumor (45), patient age (24), duration and degree of visual compromise (24), and probably also the surgical technique (25, 44). Thus, early treatment and optimal surgical planning for tuberculum sellae meningioma is very important to improve visual acuity. The ischemic, compression, or demyelination of the optic nerve are main causes of optic nerve dysfunction (46). Compression of the nerve could lead to small vessel lesions and demyelinating lesions, thus, some patients optic function might not recover even after decompression. Similarly, a partially demyelinated optic nerve may remyelinate itself after excision of the compressive mass. Previous studies about involvement of the optic canal in cases of tuberculum sellae meningiomas have all recommended opening of the optic canal and resection of the intracanalicular portion of the tumor (25, 47–49). Reviewing the literature, we can find that more than 85% of the patients with TSM who underwent optic nerve unroofing have improved and/or retained their visual acuity after surgery (13, 48–50). In our case series, eight patients performed unilateral optic canal unroofing and all of them had visual acuity improved and/or retained their visual acuity after operation. Intraoperative protection of optic nerve, separation and retention of feeding arteries of optic nerve, optic canal unroofing to relieve compression are vital for improving the visual acuity.

There are some limitations in this study. The clinical data were retrospectively collected and the results only represent the subgroup of giant tuberculum sellae meningioma patients. With regard to outcomes, if data on long-term follow-up can be acquired, the conclusion will be more accurate. Furthermore, the case series were small and acquired from a single center, and only descriptive analysis but without statistical analysis, which may lead to some inevitable bias in the conclusion.

## Conclusion

Summarize the experience of our center and previous literature, unilateral subfrontal approach can completely remove the giant sellae nodule meningioma (the maximum diameter is ≥ 30mm), and has good visual retention rate and low postoperative complication rate. It is a feasible surgical treatment for giant TSM. Intraoperative application of EC glue and pedicled fascia flap to repair the frontal sinus can significantly prevent cerebrospinal fluid leakage and frontal sinus infection. Optic canal unroofing has huge advantage in visual improvement especially for patients with visual impairment.

## Data Availability Statement

The original contributions presented in the study are included in the article/supplementary material. Further inquiries can be directed to the corresponding authors.

## Ethics Statement

The studies involving human participants were reviewed and approved by Clinical Research Ethics Committee of the First Affiliated Hospital, Zhejiang University School of Medicine. Written informed consent for participation was not required for this study in accordance with the national legislation and the institutional requirements.

## Author Contributions

FX has contributed in conceptualization, methodology, writing - review & editing and visualization. JS has contributed in writing - original draft. LZ has contributed in formal analysis and visualization. JY has contributed in visualization. YW has contributed in data curation. ZF has contributed in data curation. CZ has contributed in data curation. HY has contributed in data curation. RZ has contributed in conceptualization, validation, and supervision. XZ has contributed in conceptualization, validation, and supervision. All authors contributed to the article and approved the submitted version.

## Conflict of Interest

The authors declare that the research was conducted in the absence of any commercial or financial relationships that could be construed as a potential conflict of interest.

## Publisher’s Note

All claims expressed in this article are solely those of the authors and do not necessarily represent those of their affiliated organizations, or those of the publisher, the editors and the reviewers. Any product that may be evaluated in this article, or claim that may be made by its manufacturer, is not guaranteed or endorsed by the publisher.

## Abbreviations

mTCA, Microsurgical Transcranial approach; TSM, Tuberculum Sellae Meningiomas; CSF, Cerebrospinal fluid; EC, Ear-cerebrum; UPSIT, University of Pennsylvania Smell Identification Test; EEA, endoscopic endonasal approach; VIS, visual impairment score; GTR, gross total resection; DVI, Duration of visual impairment; VAO, Visual symptom after operation.

## References

[1] Whittle IR Smith C Navoo P Collie D. Meningiomas. Lancet (2004) 363(9420):1535–43. 10.1016/S0140-6736(04)16153-9 15135603

[2] Marosi C Hassler M Roessler K Reni M Sant M Mazza E. Meningioma. Crit Rev Oncol Hematol (2008) 67(2):153–71. 10.1016/j.critrevonc.2008.01.010 18342535

[3] de Divitiis E Esposito F Cappabianca P Cavallo L dD O. Tuberculum Sellae Meningiomas: High Route or Low Route? A Series of 51 Consecutive Cases. Neurosurgery (2008) 62:556–63. 10.1227/01.neu.0000317303.93460.24 18425005

[4] Bowers CA Altay T Couldwell WT. Surgical Decision-Making Strategies in Tuberculum Sellae Meningioma Resection. Neurosurg Focus (2011) 30(5):E1. 10.3171/2011.2.FOCUS1115 21529165

[5] Jallo GBV. Tuberculum Sellae Meningiomas: Microsurgical Anatomy and Surgical Technique. Neurosurgery (2002) 51:1432–40. 10.1097/00006123-200212000-00013 12445348

[6] Nakamura M Roser F Struck M Vorkapic P Samii M. Tuberculum Sellae Meningiomas: Clinical Outcome Considering Different Surgical Approaches. Neurosurgery (2006) 59(5):1019–28; discussion 1028–9. 10.1227/01.NEU.0000245600.92322.06 17143236

[7] Romani R Laakso A Kangasniemi M Niemela M Hernesniemi J. Lateral Supraorbital Approach Applied to Tuberculum Sellae Meningiomas: Experience With 52 Consecutive Patients. Neurosurgery (2012) 70(6):1504–18. 10.1227/NEU.0b013e31824a36e8 22240812

[8] da Silva CE de Freitas PE. Large and Giant Skull Base Meningiomas: The Role of Radical Surgical Removal. Surg Neurol Int (2015) 6:113. 10.4103/2152-7806.159489 26167365 PMC4496843

[9] Doty RL Shaman P MD. Development of the University of Pennsylvania Smell Identification Test: A Standardized Microencapsulated Test of Olfactory Function. Physiol Behav (1984) 32:489–502. 10.1016/0031-9384(84)90269-5 6463130

[10] Martin GE Junque C Juncadella M Gabarros A de Miquel MA Rubio F. Olfactory Dysfunction After Subarachnoid Hemorrhage Caused by Ruptured Aneurysms of the Anterior Communicating Artery. Clinical Article. J Neurosurg (2009) 111(5):958–62. 10.3171/2008.11.JNS08827 19361265

[11] Simpson D. The Recurrence of Intracranial Meningiomas After Surgical Treatment. J Neurol Neurosurg Psychiatry (1957) 20:22–39. 10.1136/jnnp.20.1.22 13406590 PMC497230

[12] Muskens IS Briceno V Ouwehand TL Castlen JP Gormley WB Aglio LS. The Endoscopic Endonasal Approach Is Not Superior to the Microscopic Transcranial Approach for Anterior Skull Base Meningiomas-A Meta-Analysis. Acta Neurochir (Wien) (2018) 160:59–75. 10.1007/s00701-017-3390-y 29127655 PMC5735207

[13] Chokyu I Goto T Ishibashi K Nagata T Ohata K. Bilateral Subfrontal Approach for Tuberculum Sellae Meningiomas in Long-Term Postoperative Visual Outcome. J Neurosurg (2011) 115(4):802–10. 10.3171/2011.5.JNS101812 21740117

[14] Curey S Derrey S Hannequin P Hannequin D Freger P Muraine M. Validation of the Superior Interhemispheric Approach for Tuberculum Sellae Meningioma: Clinical Article. J Neurosurg (2012) 117(6):1013–21. 10.3171/2012.9.JNS12167 23061383

[15] Terasaka S Asaoka K Kobayashi H Yamaguchi S. Anterior Interhemispheric Approach for Tuberculum Sellae Meningioma. Neurosurgery (2011) 68(1 Suppl Operative):84–8. 10.1227/NEU.0b013e31820781e1 21206321

[16] Bitter AD Stavrinou LC Ntoulias G Petridis AK Dukagjin M Scholz M. The Role of the Pterional Approach in the Surgical Treatment of Olfactory Groove Meningiomas: A 20-Year Experience. J Neurol Surg B Skull Base (2013) 74(2):97–102. 10.1055/s-0033-1333618 24436895 PMC3699215

[17] Muhammad Zafrullah A Ignatius M Sidabutar R Atmadja Wirjomartani B Faried A. Pterional Approach *Versus* Unilateral Frontal Approach on Tuberculum Sellae Meningioma: Single Centre Experiences. Asian J Neurosurg (2012) 7(1):21–4. 10.4103/1793-5482.95691 PMC3358953 22639687

[18] Reisch R Perneczky A Filippi R. Surgical Technique of the Supraorbital Key-Hole Craniotomy. Surg Neurol (2003) 59:223–7. 10.1016/S0090-3019(02)01037-6 12681560

[19] Schwartz TH Morgenstern PF Anand VK. Lessons Learned in the Evolution of Endoscopic Skull Base Surgery. J Neurosurg (2019) 130(2):337–46. 10.3171/2018.10.JNS182154 30717035

[20] Jang WY Jung S Jung TY Moon KS Kim IY. The Contralateral Subfrontal Approach can Simplify Surgery and Provide Favorable Visual Outcome in Tuberculum Sellae Meningiomas. Neurosurg Rev (2012) 35(4):601–7; discussion 607-608. 10.1007/s10143-012-0397-y 22669329

[21] Goel A Muzumdar D Desai KI. Tuberculum Sellae Meningioma: A Report on Management on the Basis of a Surgical Experience With 70 Patients. Neurosurgery (2002) 51:1358–63. 10.1097/00006123-200212000-00005 12445340

[22] Benjamin V Russell SM. The Microsurgical Nuances of Resecting Tuberculum Sellae Meningiomas. Neurosurgery (2005) 56(2 Suppl):411–7. 10.1227/01.NEU.0000144783.07688.BC 15794838

[23] Fahlbusch R Schott W. Pterional Surgery of Meningiomas of the Tuberculum Sellae and Planum Sphenoidale: Surgical Results With Special Consideration of Ophthalmological and Endocrinological Outcomes. J Neurosurg (2002) 96:235–43. 10.3171/jns.2002.96.2.0235 11838796

[24] Pamir MN Ozduman K Belirgen M Kilic T Ozek MM. Outcome Determinants of Pterional Surgery for Tuberculum Sellae Meningiomas. Acta Neurochir (Wien) (2005) 147(11):1121–30; discussion 1130. 10.1007/s00701-005-0625-0 16133766

[25] Mathiesen T Kihlstrom L. Visual Outcome of Tuberculum Sellae Meningiomas After Extradural Optic Nerve Decompression. Neurosurgery (2006) 59(3):570–6. 10.1227/01.NEU.0000228683.79123.F9 16955039

[26] Park CK Jung HW Yang SY Seol HJ Paek SH Kim DG. Surgically Treated Tuberculum Sellae and Diaphragm Sellae Meningiomas: The Importance of Short-Term Visual Outcome. Neurosurgery (2006) 59(2):238–43. 10.1227/01.NEU.0000223341.08402.C5 16883164

[27] Diraz A Kobayashi S Toriyama T Ohsawa M Hokama M Kitazama K. Surgical Approaches to the Anterior Communicating Artery Aneurysm and Their Results. Neurol Res (1993) 15:273–80. 10.1080/01616412.1993.11740148 8105408

[28] Ganna A Dehdashti AR Karabatsou K Gentili F. Fronto-Basal Interhemispheric Approach for Tuberculum Sellae Meningiomas; Long-Term Visual Outcome. Br J Neurosurg (2009) 23(4):422–30. 10.1080/02688690902968836 19637015

[29] Attia M Kandasamy J Jakimovski D Bedrosian J Alimi M Lee DL. The Importance and Timing of Optic Canal Exploration and Decompression During Endoscopic Endonasal Resection of Tuberculum Sella and Planum Sphenoidale Meningiomas. Neurosurgery (2012) 71(1 Suppl Operative):58–67. 10.1227/NEU.0b013e318258e23d 22517253

[30] Khan OH Anand VK Schwartz TH. Endoscopic Endonasal Resection of Skull Base Meningiomas: The Significance of a “Cortical Cuff” and Brain Edema Compared With Careful Case Selection and Surgical Experience in Predicting Morbidity and Extent of Resection. Neurosurg Focus (2014) 37(4):E7. 10.3171/2014.7.FOCUS14321 25465040

[31] Laufer I Anand V Schwartz T. Endoscopic, Endonasal Extended Transsphenoidal, Transplanum Transtuberculum Approach for Resection of Suprasellar Lesions. J Neurosurg (2007) 106(3):400–6. 10.3171/jns.2007.106.3.400 17367062

[32] Mascarenhas L Moshel YA Bayad F Szentirmai O Salek AA Leng LZ. The Transplanum Transtuberculum Approaches for Suprasellar and Sellar-Suprasellar Lesions: Avoidance of Cerebrospinal Fluid Leak and Lessons Learned. World Neurosurg (2014) 82(1-2):186–95. 10.1016/j.wneu.2013.02.032 23403355

[33] Song SW Kim YH Kim JW Park CK Kim JE Kim DG. Outcomes After Transcranial and Endoscopic Endonasal Approach for Tuberculum Meningiomas-A Retrospective Comparison. World Neurosurg (2018) 109:e434–45. 10.1016/j.wneu.2017.09.202 29017976

[34] Bander ED Singh H Ogilvie CB Cusic RC Pisapia DJ Tsiouris AJ. Endoscopic Endonasal *Versus* Transcranial Approach to Tuberculum Sellae and Planum Sphenoidale Meningiomas in a Similar Cohort of Patients. J Neurosurg (2018) 128(1):40–8. 10.3171/2016.9.JNS16823 28128693

[35] Komotar RJ Starke RM Raper DM Anand VK Schwartz TH. Endoscopic Endonasal *Versus* Open Transcranial Resection of Anterior Midline Skull Base Meningiomas. World Neurosurg (2012) 77(5-6):713–24. 10.1016/j.wneu.2011.08.025 22120296

[36] Kong DS Hong CK Hong SD Nam DH Lee JI Seol HJ. Selection of Endoscopic or Transcranial Surgery for Tuberculum Sellae Meningiomas According to Specific Anatomical Features: A Retrospective Multicenter Analysis (KOSEN-002). J Neurosurg (2018) 130(3):838–47. 10.3171/2017.11.JNS171337 29775151

[37] Hadad G Bassagasteguy L Carrau RL Mataza JC Kassam A Snyderman CH. A Novel Reconstructive Technique After Endoscopic Expanded Endonasal Approaches: Vascular Pedicle Nasoseptal Flap. Laryngoscope (2006) 116(10):1882–6. 10.1097/01.mlg.0000234933.37779.e4 17003708

[38] Ottenhausen M Banu MA Placantonakis DG Tsiouris AJ Khan OH Anand VK. Endoscopic Endonasal Resection of Suprasellar Meningiomas: The Importance of Case Selection and Experience in Determining Extent of Resection, Visual Improvement, and Complications. World Neurosurg (2014) 82(3-4):442–9. 10.1016/j.wneu.2014.03.032 24657254

[39] Black P. Cerebrospinal Fluid Leaks Following Spinal or Posterior Fossa Surgery: Use of Fat Grafts for Prevention and Repair. Neurosurg Focus (2000) 9(1):e4. 10.3171/foc.2000.9.1.4 16865811

[40] Weber R Draf W Keerl R Kahle G Schinzel S Thomann S. Osteoplastic Frontal Sinus Surgery With Fat Obliteration: Technique and Long-Term Results Using Magnetic Resonance Imaging in 82 Operations. Laryngoscope (2000) 110(6):1037–44. 10.1097/00005537-200006000-00028 10852527

[41] Elia AE Shih HA Loeffler JS. Stereotactic Radiation Treatment for Benign Meningiomas. Neurosurg Focus (2007) 23(4):E5. 10.3171/FOC-07/10/E5 17961042

[42] Di Maio S Ramanathan D Garcia-Lopez R Rocha MH Guerrero FP Ferreira M. Evolution and Future of Skull Base Surgery: The Paradigm of Skull Base Meningiomas. World Neurosurg (2012) 78(3-4):260–75. 10.1016/j.wneu.2011.09.004 22120278

[43] Minniti G Amichetti M Enrici RM. Radiotherapy and Radiosurgery for Benign Skull Base Meningiomas. Radiat Oncol (2009) 4:42. 10.1186/1748-717X-4-42 19828022 PMC2768735

[44] Kitano M Taneda M Nakao Y. Postoperative Improvement in Visual Function in Patients With Tuberculum Sellae Meningiomas: Results of the Extended Transsphenoidal and Transcranial Approaches. J Neurosurg (2007) 107(2):337–46. 10.3171/JNS-07/08/0337 17695388

[45] Schick U Hassler W. Surgical Management of Tuberculum Sellae Meningiomas: Involvement of the Optic Canal and Visual Outcome. J Neurol Neurosurg Psychiatry (2005) 76(7):977–83. 10.1136/jnnp.2004.039974 PMC1739719 15965205

[46] Lee JH Jeun SS Evans J Kosmorsky G. Surgical Management of Clinoidal Meningiomas. Neurosurgery (2001) 48(5):1012–9. 10.1227/00006123-200105000-00009 11334267

[47] Sade B Lee JH. High Incidence of Optic Canal Involvement in Tuberculum Sellae Meningiomas: Rationale for Aggressive Skull Base Approach. Surg Neurol (2009) 72(2):118–23. 10.1016/j.surneu.2008.08.007 19147207

[48] Mahmoud M Nader R Al-Mefty O. Optic Canal Involvement in Tuberculum Sellae Meningiomas: Influence on Approach, Recurrence, and Visual Recovery. Neurosurgery (2010) 67(3 Suppl Operative):ons108–19. 10.1227/01.NEU.0000383153.75695.24 20679940

[49] Nozaki K Kikuta K-I Takagi Y Mineharu Y Takahashi JA Hashimoto N. Effect of Early Optic Canal Unroofing on the Outcome of Visual Functions in Surgery for Meningiomas of the Tuberculum Sellae and Planum Sphenoidale. Neurosurgery (2008) 62(4):839–46. 10.1227/01.neu.0000318169.75095.cb 18496190

[50] Attia M Umansky F Paldor I Dotan S Shoshan Y Spektor S. Giant Anterior Clinoidal Meningiomas: Surgical Technique and Outcomes. J Neurosurg (2012) 117(4):654–65. 10.3171/2012.7.JNS111675 22900847

